# Supplementary material for: Intracellular tension sensor reveals mechanical anisotropy of the actin cytoskeleton
Source: Nat Commun. 2023 Dec 4;14:8011. doi: 10.1038/s41467-023-43612-5 (PMC10695988; doi:10.1038/s41467-023-43612-5)
Supplement: Supplementary file 1 — Supplementary Information [file 41467_2023_43612_MOESM1_ESM.pdf]

Intracellular Tension Sensor Reveals Mechanical Anisotropy of the Actin Cytoskeleton

Sorosh Amiri[1,2], Camelia Muresan[1,3], Xingbo Shang[1,3], Clotilde Huet-Calderwood[4], Martin A Schwartz[3,5,6], David A Calderwood[4,5], Michael Murrell [1,3,7,*]

[1]Systems Biology Institute, 850 West Campus Drive, Yale University, West Haven, CT, 06516, USA

[2]Department of Mechanical Engineering and Material Science, 17 Hillhouse Ave, Yale University, New Haven, CT, 06511, USA

[3]Department of Biomedical Engineering, 17 Hillhouse Ave, Yale University, New Haven, CT, 06511, USA

[4]Department of Pharmacology, 333 Cedar St, Yale University, New Haven, CT 06510, USA

[5]Department of Cell Biology, 333 Cedar St, Yale University, New Haven, CT 06510, USA

[6]Yale Cardiovascular Research Center, 300 George St, New Haven, CT 06511, USA

[7] Department of Physics, 217 Prospect Street, Yale University, New Haven, CT, 06511, USA

SUPPLEMENTARY INFORMATION

- Supplementary Notes
- Supplementary Figures
- Supplementary Videos

Supplementary Notes 1: Estimation of the internal stress from traction force microscopy data

To estimate the internal stresses from the Traction Force Microscopy (TFM) method, we assumed cell is a thin homogenous, isotropic, linear elastic disk (HIE assumptions) attached to the substrate through peripheral focal adhesions. Therefore, the force balance equations, and Beltrami-Mitchell compatibility equations[1,2] can be written as:

$$\frac{-T_x}{h} = \frac{\partial \sigma_x}{\partial x} + \frac{\partial \sigma_{xy}}{\partial y}$$

$$\frac{-T_y}{h} = \frac{\partial \sigma_y}{\partial y} + \frac{\partial \sigma_{xy}}{\partial x}$$

$$\left(\frac{\partial^2}{\partial x^2} + \frac{\partial^2}{\partial y^2}\right)(\sigma_x + \sigma_y) = \frac{-1}{h}(1+\nu)\left(\frac{\partial T_x}{\partial x} + \frac{\partial T_y}{\partial y}\right)$$

Where T_x and T_y are the x and y components of the traction stress vectors applied to the substrate by cells, h=5 µm is the cell thickness and ν is the Poisson ratio. Solving for σ_x, σ_y and σ_{xy} will give the internal stress. The average normal stress was then calculated by:

$$|\tilde{\sigma}| = \frac{\sigma_x + \sigma_y}{2}$$

And the average internal force was calculated by $\left|\tilde{F}\right| = |\tilde{\sigma}| \times A$, where A is the area over which the inferred internal stresses were calculated.

Supplementary Notes 2: Calculation of the Relative FRET change

We used the relative FRET change averaged over each cell or over stress fibers to account for intracellular changes. The relative FRET change was calculated as following:

$$\Delta\text{FRET index} = \text{Relative FRET index change} = \frac{(FRETi_s - FRETi_r)}{FRETi_r}$$

$$\Delta\text{FRET E} = \text{Relative FRET E change} = \frac{(FRET\ E_s - FRET\ E_r)}{FRET\ E_r}$$

$$\Delta\text{RFP} = \text{Relative RFP change} = \frac{(RFP_s - RFP_r)}{RFP_r}$$

$$\Delta\text{RFP/A} = \text{Relative RFP/A change} = \frac{(RFP/A_s - RFP/A_r)}{RFP/A_r}$$

Where RFP is the integrated fluorescent intensity of the sensor, and RFP/A is the density of the sensor fluorescent.

Supplementary Table 1

Primers used for QuickChange® and Gibson assembly of intermolecular FRET and FRET efficiency controls.

**TS-RFP***	
**RFPdark_CT_Forward**	ACCAGCTTCATGCTCGGCAGCAGAAC
**RFPdark_CT_Reverse**	GTTCTGCTGCCGAGCATGAAGCTGGT
**TS-GFP***	
**GFP_CT-Forward**	CGTGACCACCCTGACCCTCGGCGTGCAGTGCTTC
**GFP_CT-Reverse**	GAAGCACTGCACGCCGAGGGTCAGGGTGGTCACG
**Short Linker (GGSGGS)$_2$**	
**4XGGS_Forward**	ggaggatccggaggatccATGGTGTCTAAGGGCGAAGAGCTGATTAAGG
**4XGGS_Reverse**	ggatcctccggatcctccCCCGGCGGCGGTCACGAA
**4XGGS_Check**	CCACAACATCGAGGACGGCAGC
**Long Linker (TRAF)**	
**TS_F**	actcagatctATGGTGTCTAAGGGCGAAGAGCTGATTAAGG
**TS_R**	tctctccggaCCCGGCGGCGGTCACGAA
**TRAF_F**	cgccgccgggTCCGGAGAGAGCCTGGAG
**TRAF_R**	tagacaccatAGATCTGAGTCCGGAGAGC
**GFP-(GGSGGS)$_2$/TRAF2TRAF-RFP_Fwd**	gcttggtaccatggtgagcaagggcgag
**GFP-(GGSGGS)$_2$/TRAF2TRAF-RFP_Rev**	ccgcttattaattaagtttgtgccccagtttg
**pcDNA3_Fwd**	caaacttaattaataagcggccgctcgag
**pcDNA3_Rev**	tgctcaccatggtaccaagcttgggtctc
**pcDNA3_check_Fwd**	cgcaaatgggcggtaggcgtg
**pcDNA3_check_Rev**	tggctggcaactagaaggcaca

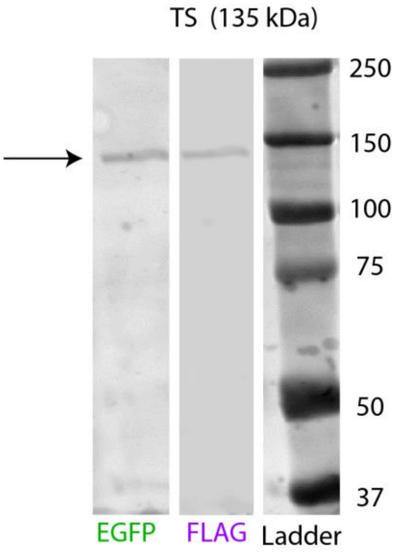

Supplementary Figure 1. Western blot and correlation analysis showing expression of TS. Whole cell lysates 2 days after transfection expressing TS were separated on 10% SDS-PAGE and transferred to nitrocellulose membrane. Polyclonal anti-EGFP antibody detected a single band with an apparent molecular mass of 135 kDa. A polyclonal anti-FLAG antibody recognizing the C terminus of TS detected a single band with an apparent molecular mass of 135 kDa.

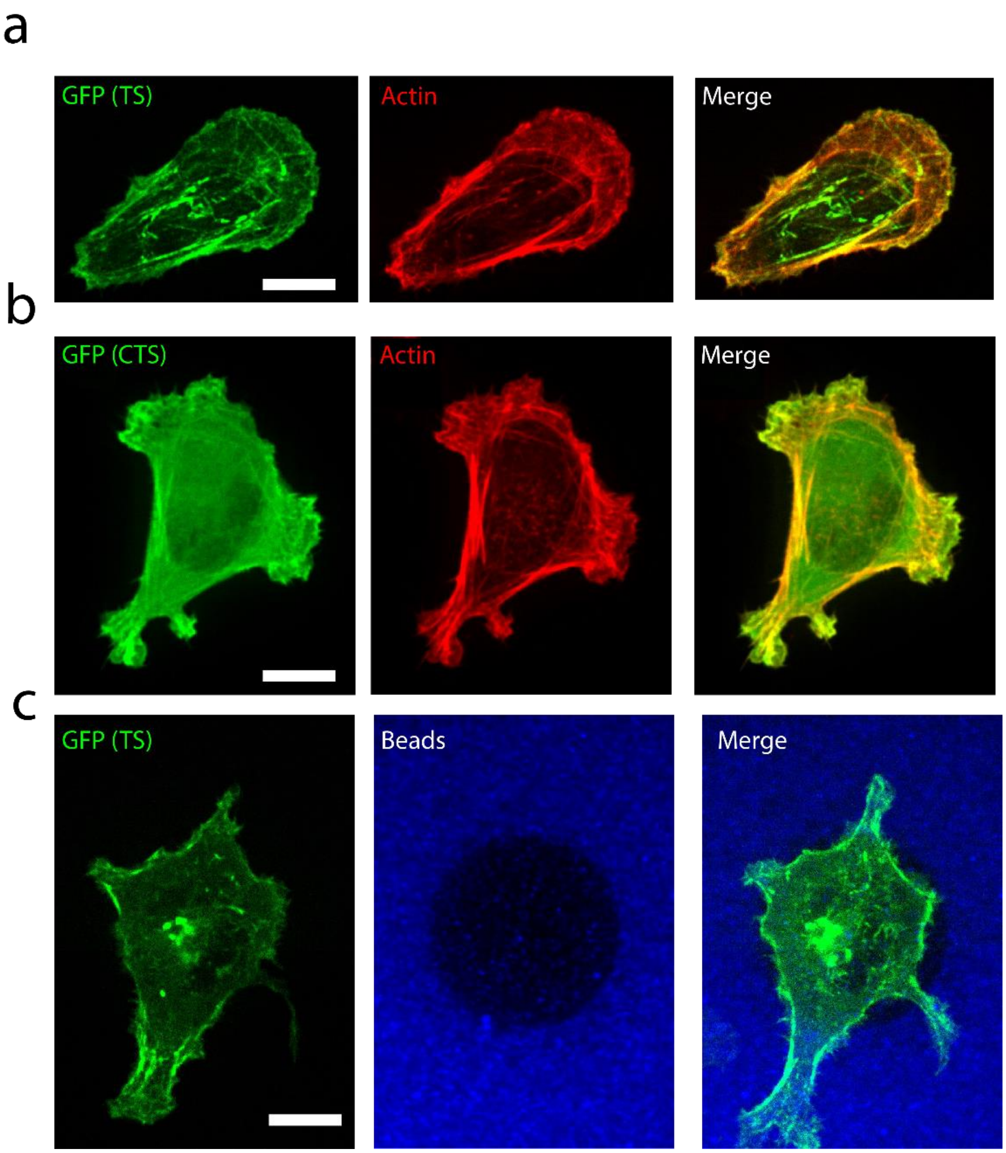

Supplementary Figure 2. Unpatterned and Y27632 treated TS and CTS cells. (a) Immunofluorescent phalloidin staining of unpatterned TS and (b) CTS cells. Scale bar is 20 µm. (c) ROCK inhibitor, Y27632 treated TS cell on a circular pattern. Scale bar is 10 µm.

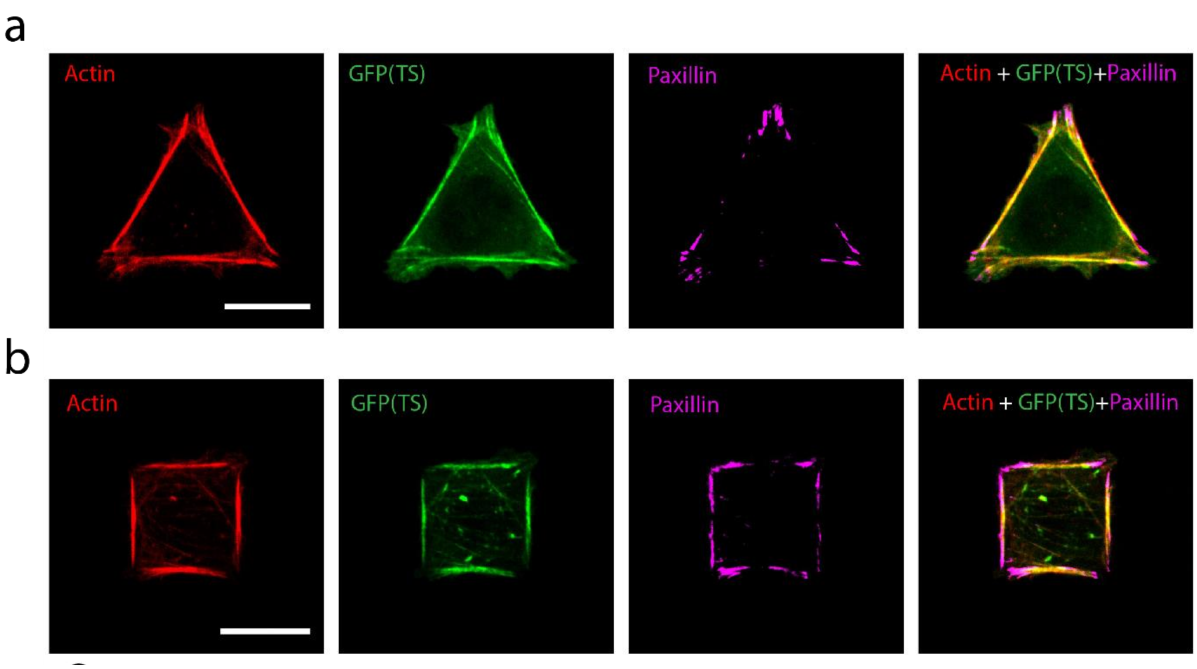

Supplementary Figure 3. Immunofluorescent phalloidin staining of triangle and square shaped TS and CTS cells. (a) GFP, phalloidin actin, paxillin and merged imaged of TS expressing U2OS cells on triangular patterns. (b) GFP, phalloidin actin, paxillin and merged imaged of CTS expressing U2OS cells on square patterns. Scale bar is 20 μm.

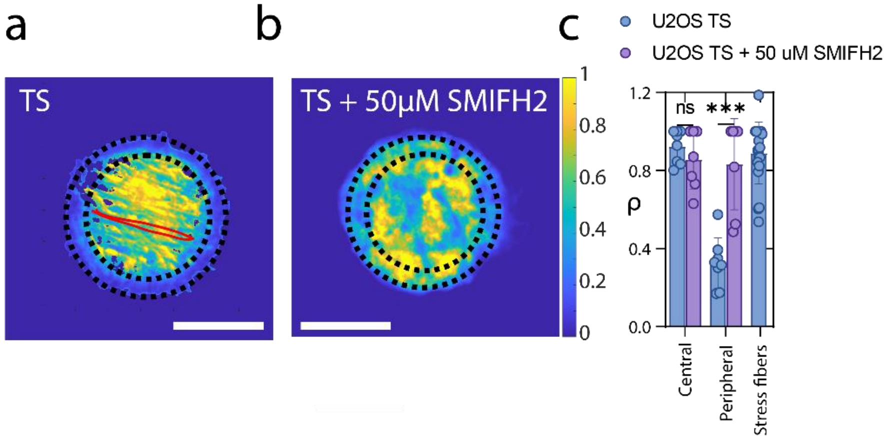

Supplementary Figure 4. Thresholded sensor enrichment (ρ). (a) Sensor enrichment in TS cell omitting regions with low 30% sensor concentration (b) Sensor enrichment in TS+50μM SMIFH2 cell omitting regions with low 30% sensor concentration (c) Sensor enrichment (ρ) in the central, peripheral and SFs regions (n=10 for U2OS TS cells, n=26 for SFs, n=13 for U2OS TS+SMIFH2 cells. CK666 treated cells showed enrichments comparable to non-treated cells, explainable by formation of branched actin in the presence of the TS sensor. T-tests were used for significance. p(central)=0.305, p(peripheral)=0.00014. Scale bar is 10 μm. Source data are provided as a Source Data file.

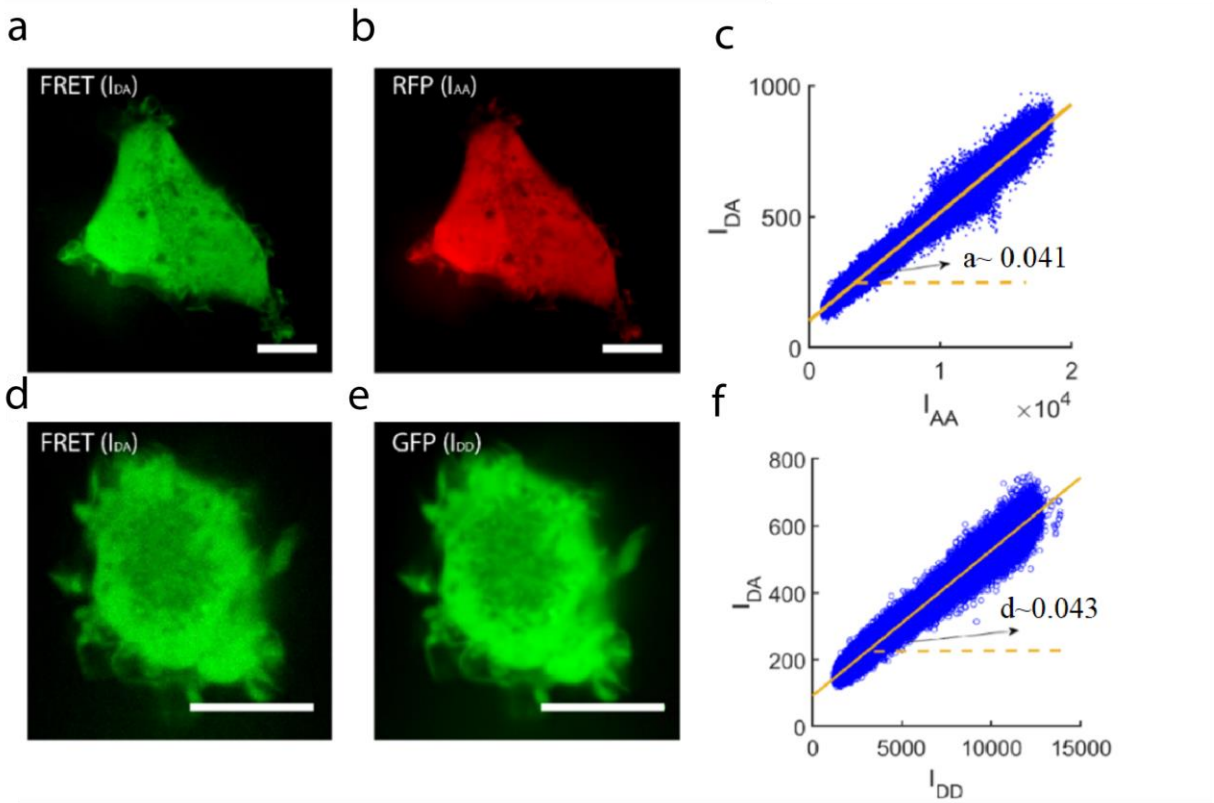

Supplementary Figure 5. Bleed through analysis for FRET calculation. (a) FRET channel of a RFP transfected cell (b) 560nm channel of a RFP cell. Scale bars 5 µm. (c) correlation of FRET vs 560nm image, gives the slope for bleed through analysis. (d) FRET channel of a GFP transfected cell. Scale bars 10 µm. (e) 480nm channel of a GFP transfected cell (f) correlation of FRET vs 560nm image, gives the slope for bleed through analysis. Regression lines with p-value<0.0001.

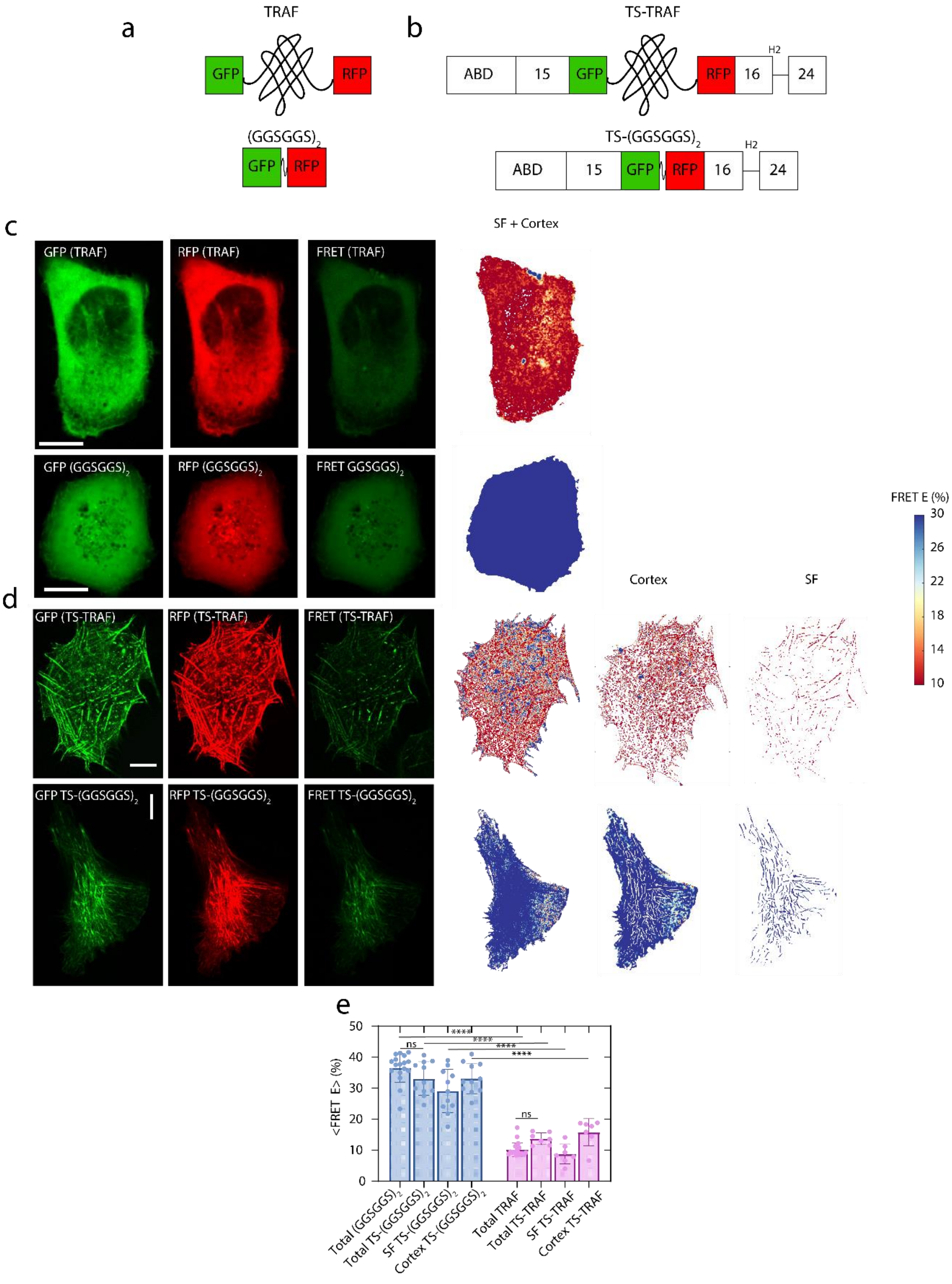

Supplementary Figure 6. FRET efficiency measurement controls. (a) Fluorophore bound long linker TRAF, and short linker $(GGSGGS)_2$ construct. (b) TS with long linker TS-TRAF and TS with short linker TS-$(GGSGGS)_2$ construct. (c) GFP, RFP and FRET channel of a TRAF expressing cell and the corresponding FRET Efficiency for the total area, and GFP, RFP and FRET channel of a $(GGSGGS)_2$ expressing cell and the corresponding FRET Efficiency for the total area. Scale bar is 10 µm. (d) GFP, RFP and FRET channel of a TS-TRAF cell and the corresponding FRET Efficiency for the

total, SF and cortex of cell, and GFP, RFP and FRET channel of a TS-$(GGSGGS)_2$ cell and the corresponding FRET Efficiency for the total, SF and cortex of cell. Scale bar is 5 µm. (e) FRET efficiency (FRET E) of long versus short linker expressing cells, for the total, SF and cortex regions. Šídák's significance test was conducted with **** for P<0.0001. (n=11 for TS-$(GGSGGS)_2$, n=17 for $(GGSGGS)_2$, n=7 for TS-TRAF, n=16 for TRAF). p(total (GGSGGS)2 & total TS-(GGSGGS)2)=0.7455 and p(total TRAF & total TS-TRAF)=0.9318. Source data are provided as a Source Data file.

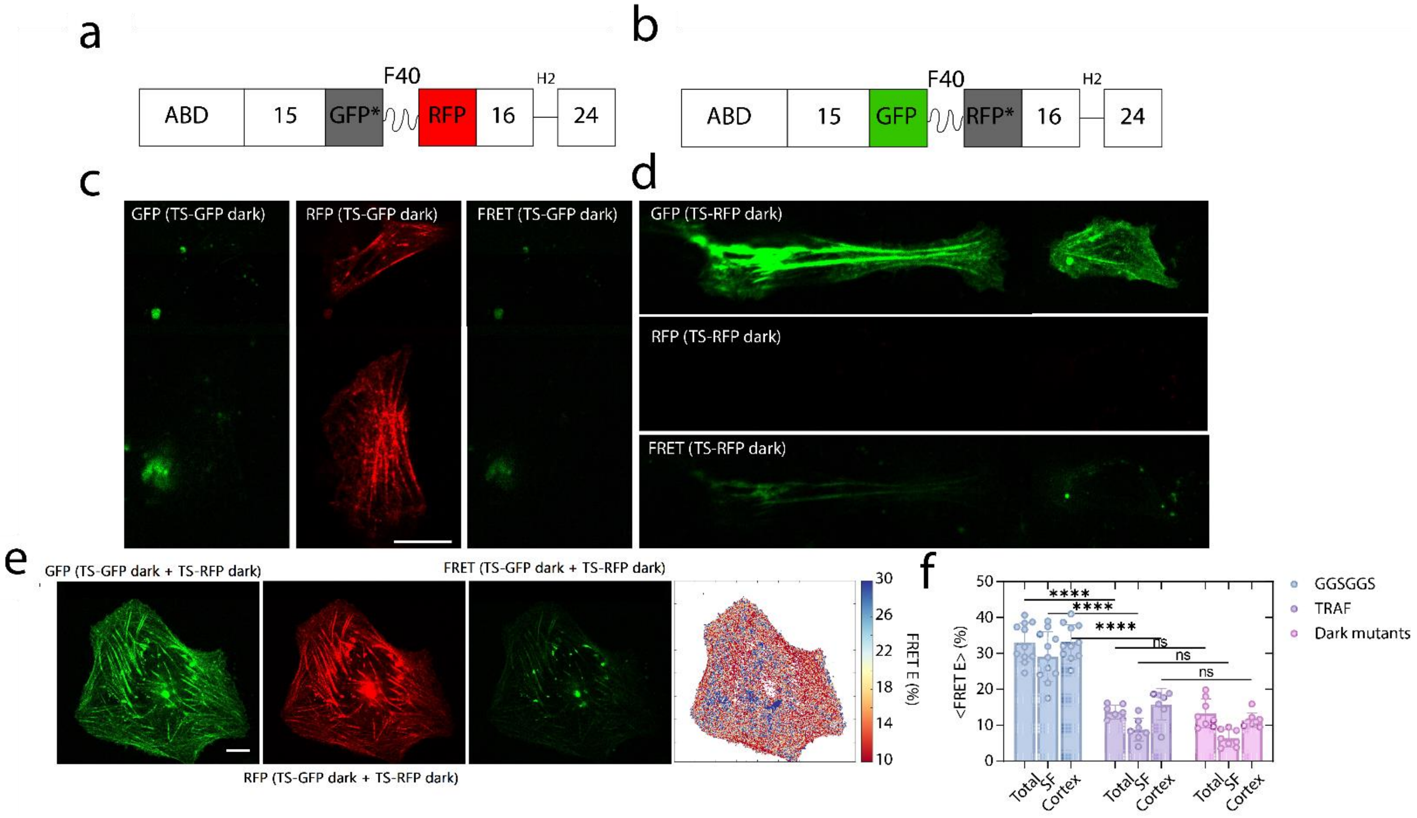

Supplementary Figure 7. Inter/intradimeric FRET control. (a) RFP-dark mutated TS construct. (b) GFP-dark mutated TS construct. (c) GFP, RFP and FRET channels of TS-GFP dark. Scale bar is 20 µm. (d) GFP, RFP and FRET channels of TS-RFP dark. (e) GFP, RFP and FRET channel of co-expressed TS-GFP dark and TS-RFP, and the corresponding FRET efficiency (FRET E). Scale bar is 5 µm. (f) FRET efficiency (FRET E) of TS-$(GGSGGS)_2$, TS-TRAF and inter/intradimeric FRET control. Tukey's significance test was conducted with **** for P<0.0001. For totals, p(TRAF & Dark mutants)=0.9783, for SFs, p(TRAF & Dark mutants)=0.5556, for cortex, p(TRAF & Dark mutants)=0.1532. (n=11 for GGSGGS, n=7 for TRAF, n=8 for dark mutants). Source data are provided as a Source Data file.

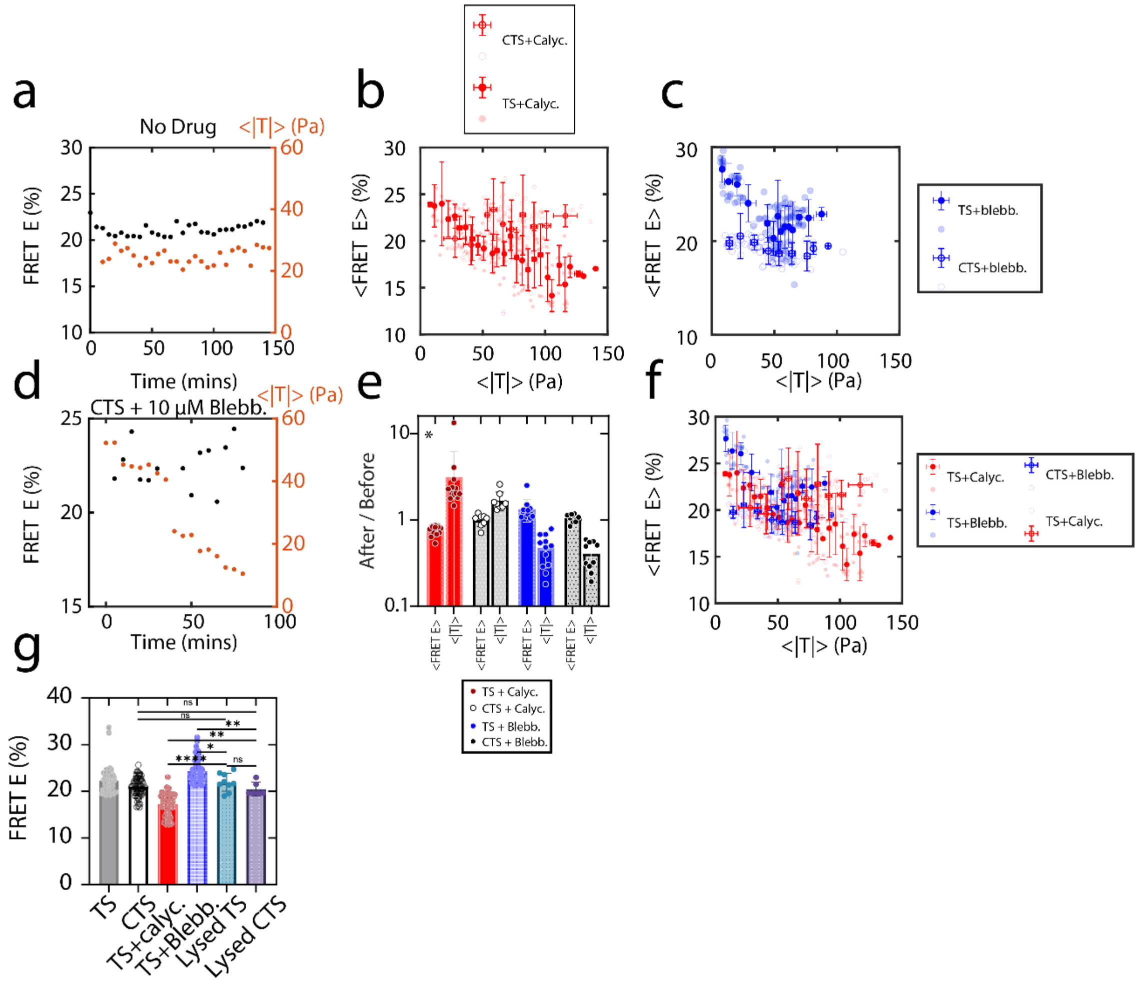

Supplementary Figure 8. **Comparison of FRET E for different drug treatments in TS and CTS cells.** (a) Average FRET E and <|T|> for no drug treated TS cell over time. (b) <FRET E>-<|T|> for Calyculin-A treated TS and CTS cells. For TS, n=12 cells N=77 measurements at different times, p-value<0.0001, for CTS n=12, ns regression with p-value=0.1579. (c) <FRET E>-<|T|> for Blebbistatin treated TS (n=7 cells N=87 measurements at different times) and CTS cells (n=7, ns regression p-value=0.1192). (d) <FRET E> and <|T|> for 10 μM Blebbistatin treated CTS cell over time. (e) Change in FRET E for CTS and TS cells before and after addition of cytoskeleton force regulating drugs. n=13 for TS+calyc., n=8 for CTS+ calyc.,n=12 for TS+Blebb., n=10 for CTS+ Blebb. 2way ANOVA significance test. p-value=0.0207 (f) overlay of <FRET E>-<|T|> data. Statistics according to panels B and C. (g) FRET E for lysed TS and lysed CTS cells compared to TS, CTS, TS+calyc and TS+Blebb cells. n=53 for CTS, n=77 for TS, n=87 for TS+calyc, n=84 for TS+Blebb., n=9 for lysed TS and n=6 for lysed CTS. Tukey's significance test was used. p(lysed CTS & lysed TS)=0.8768, p(lysed CTS & TS+calyc)<0.0001, p(lysed CTS & TS+blebb)=0.0014, p(lysed TS & TS+blebb)=0.0372, p(lysed TS & TS+calyc)<0.0001, p(lysed TS & CTS)=0.993, p(lysed CTS & CTS)=0.5083. Source data are provided as a Source Data file.

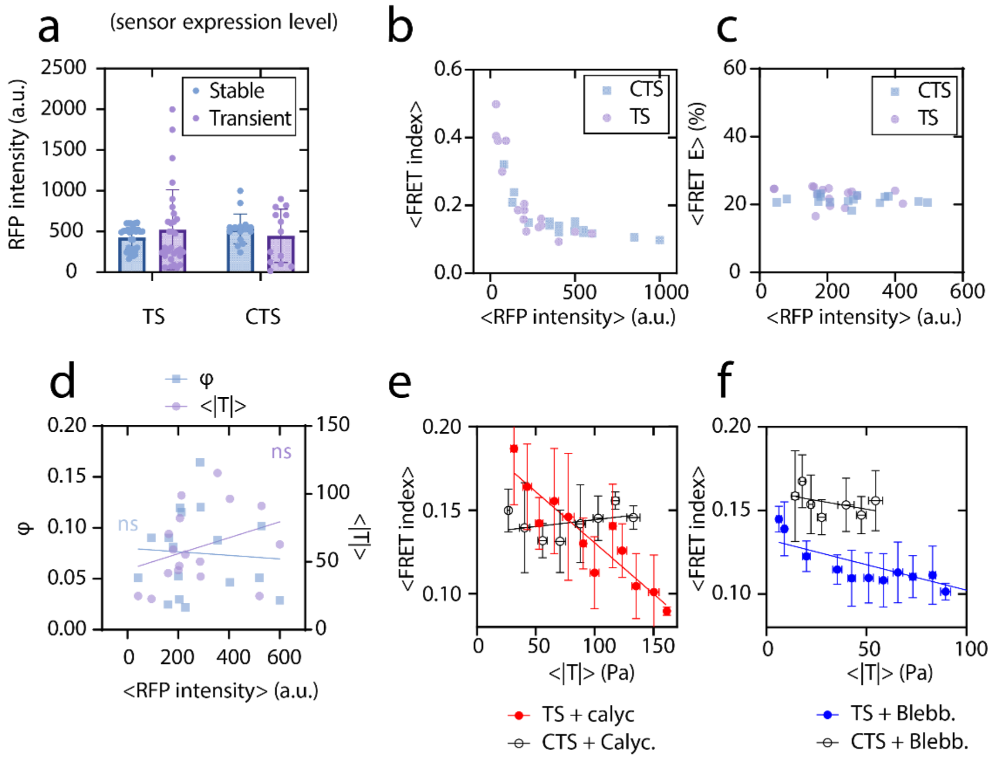

Supplementary Figure 9. Sensor expression levels correlation to FRET index and FRET efficiency. (a) Sensor expression level - RFP intensity for stably and transiently transfected TS and CTS cells. n=29 for stable TS, n=30 for transient TS, n=16 for stable CTS, n=13 for transient CTS (b) <FRET index> vs <RFP intensity> for TS and CTS cells. (c) <FRET E> vs <RFP intensity> for TS and CTS cells. (d) Mean traction stress <|T|>, and alignment order (φ) as a function of sensor expression. Non-significant slope for the regression lines p-value=0.798 for φ and p-value=0.1987 for <|T|> (e) FRET index, <|T|> relationship for TS+ calyc (p-value<0.0001), CTS+calyc, (p-value=0.1266) and (f) for TS+Blebb (p-value<0.0001). CTS+blebb (p-value=0.3561). Source data are provided as a Source Data file.

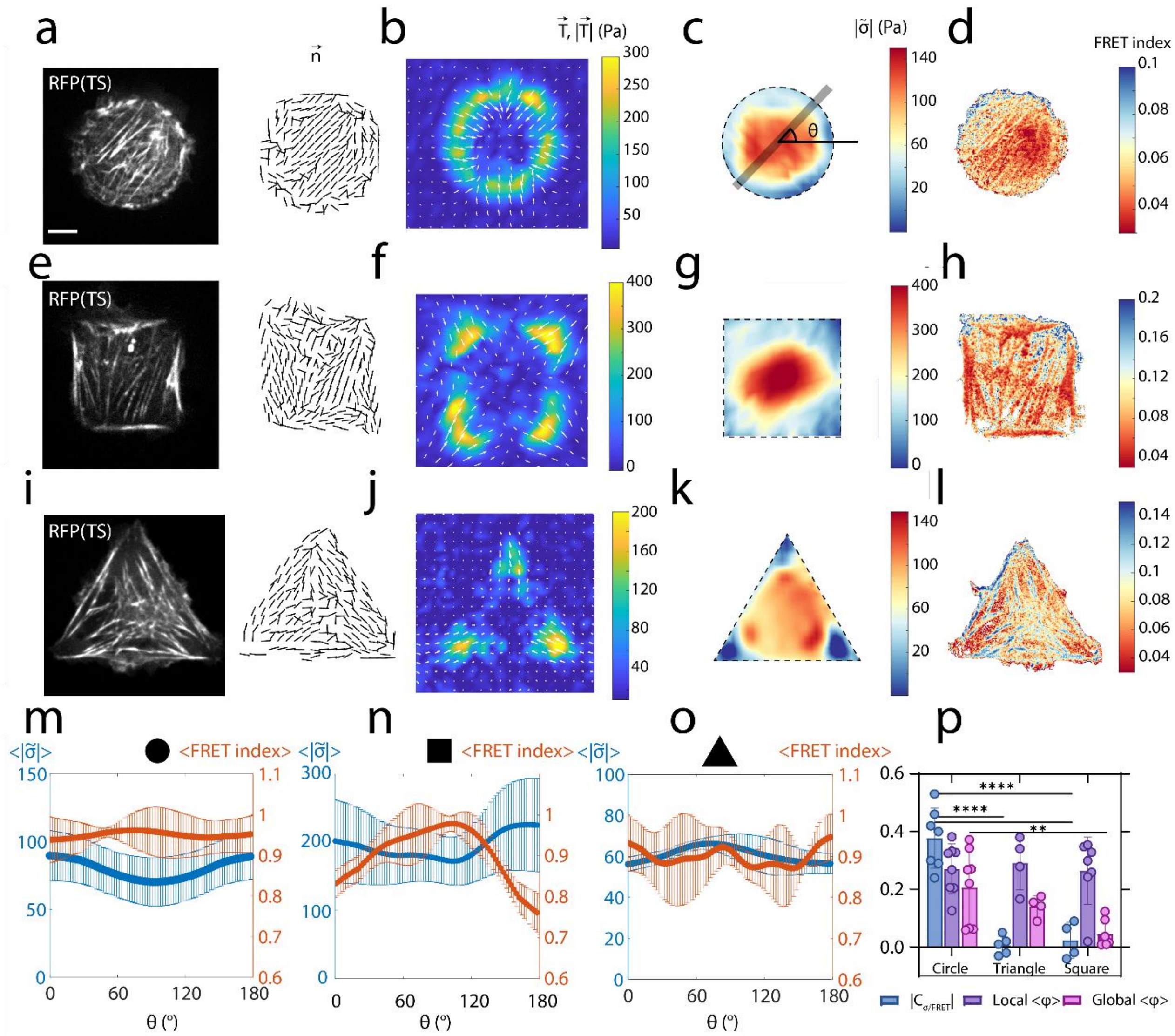

Supplementary Figure 10. FRET index representation of micropatterned cells. (a) RFP of a circular U2OS TS cell and the corresponding alignment field $\vec{\mathbf{n}}$ (b) The magnitude and vectors of traction stresses. (c) The average internal normal stress $|\tilde{\sigma}|$ calculated from the TFM stress based on linear elasticity and force balance. (d) The FRET index of circular cell. (e) RFP of a square U2OS TS cell and the corresponding alignment field $\vec{\mathbf{n}}$ (f) The magnitude and vectors of traction stresses. (g) The average internal normal stress $|\tilde{\sigma}|$ (h) The FRET index of circular cell. (i) RFP of a triangle U2OS TS cell and the corresponding alignment field $\vec{\mathbf{n}}$ (j) The magnitude and vectors of traction stresses (k) The average internal normal stress $|\tilde{\sigma}|$ (l) The FRET index of circular cell. (m) Radially averaged $|\tilde{\sigma}|$ (blue dots) and <FRET> (orange dots) as a function of θ, the angle from the center of circle, square (n), and (o) triangle shapes. (p) $C_{|\tilde{\sigma}|/\mathrm{FRET}}$, local $<\varphi>$ and global $<\varphi>$ for circle, square and triangle shapes. (n=7 for circles, n=6 for square and n=4 for triangles). Tukey's significance test was used. **** is P-value<0.0001, ** is P-value=0.004. Error bars are standard deviations from the mean. Scale bar is 5 μm. Source data are provided as a Source Data file.

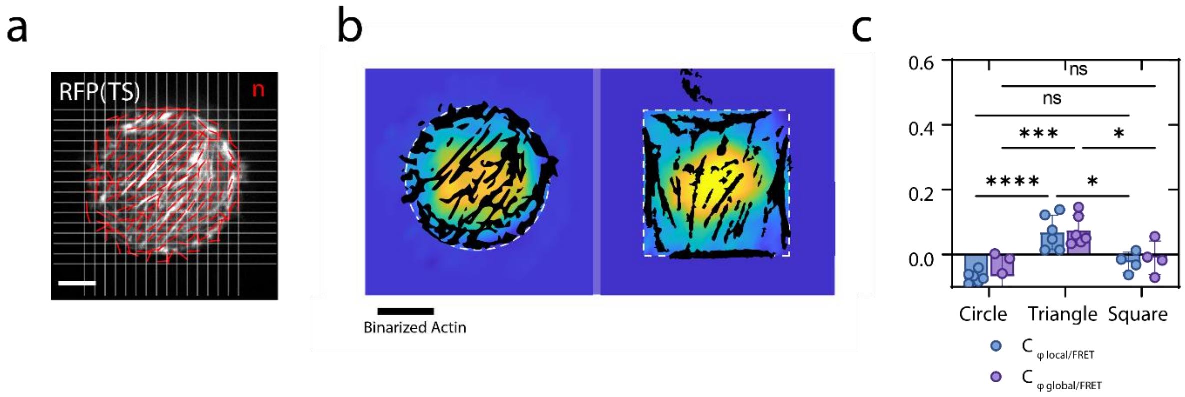

Supplementary Figure 11. **Alignment order parameter calculation**. (a) Actin alignment vectors overlayed on circular cell, and the grid over which the alignment order parameter was calculated. Scale bar is 5 µm. (b) Stress fibers and σ for circle and square shapes. (c) Spatial correlation between the FRET index and alignment order (local and global) for circle (n=6), triangle (n=6), and square (n=4) shapes. Two-way ANOVA statistical test. ** is p-value<0.01, ns is non-significant. Sidak's significance test was used. for C(φ local / FRET), p(circle & triangle)=0.0064, p(circle & square)=0.2274, p(triangle & square)=0.0150. for C(φ global / FRET), p(circle & triangle)<0.0001, p(circle & square)=0.2244, p(triangle & square)=0.0284. Source data are provided as a Source Data file.

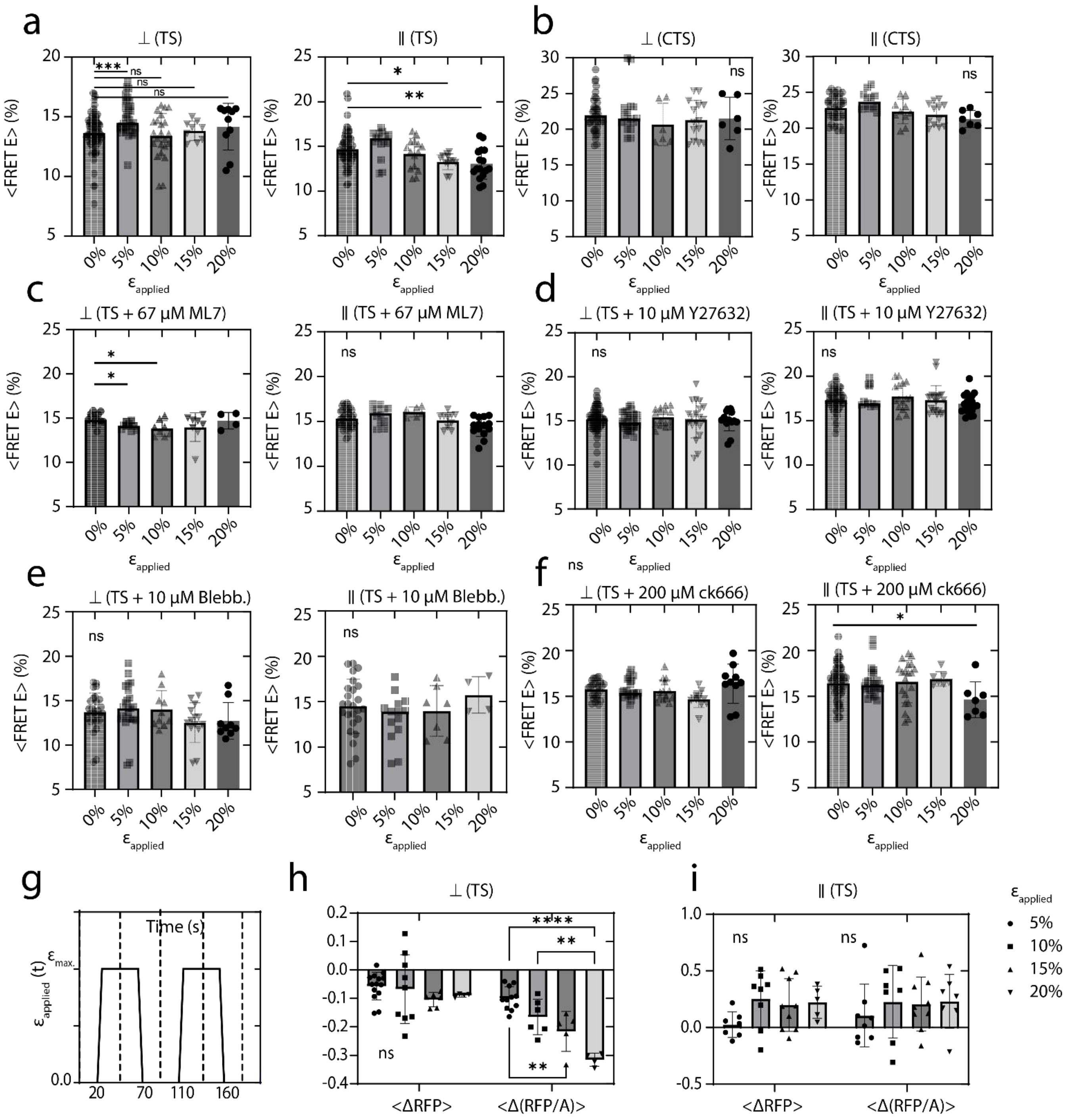

Supplementary Figure 12. **Absolute FRET E and ΔRFP values for different strains.** (A) left, <FRET E> of TS cells as a function of strain for orthogonal cells. n=78 for 0%, n=35 for 5%, n=23 for 10%, n=9 for 15%, n=10 for 20%. Dunnett's multiple tests, p(0% & 5%)=0.0005, p(0% & 10%)=0.9504, p(0% & 15%)=0.9952, p(0% & 20%)=0.7874. Right, <FRET E> of TS cells as a function of strain for parallel cells. n=63 for 0%, n=15 for 5%, n=15 for 10%, n=9 for 15%, n=16 for 20%. Dunnett's multiple tests, p(0% & 5%)=0.5393, p(0% & 10%)=0.7349, p(0% & 15%)=0.0409, p(0% & 20%)=0.0036. (B) left, <FRET E> of CTS cells as a function of strain for orthogonal cells. n=43 for 0%, n=16 for 5%, n=6 for 10%, n=14 for 15%, n=6 for 20%. Dunnett's multiple tests, p(0% & 5%)= 0.9860, p(0% & 10%)=0.7064, p(0% & 15%)=0.8835, p(0% & 20%)=0.9908. Right, <FRET E> of CTS cells as a function of strain for parallel cells. n=63 for 0%, n=15 for 5%, n=15

for 10%, n=9 for 15%, n=16 for 20%. Dunnett's multiple tests, p(0% & 5%)=0.1954, p(0% & 10%)=0.8449, p(0% & 15%)=0.2881, p(0% & 20%)=0.0823 (C) left, <FRET E> of ML7 treated TS cells as a function of strain for orthogonal cells. n=14 for 0%, n=10 for 5%, n=8 for 10%, n=8 for 15%, n=4 for 20%. Dunnett's multiple tests, p(0% & 5%)=0.0424, p(0% & 10%)=0.0181, p(0% & 20%)=0.894. Right, <FRET E> of ML7 treated TS cells as a function of strain for parallel cells. n=43 for 0%, n=12 for 5%, n=6 for 10%, n=10 for 15%, n=14 for 20%. Dunnett's multiple tests, p(0% & 5%)=0.625, p(0% & 10%)=0.294, p(0% & 15%)=0.967. (D) left, <FRET E> of Y27632 treated TS cells as a function of strain for orthogonal cells. n=64 for 0%, n=34 for 5%, n=16 for 10%, n=11 for 15%, n=14 for 20%. Dunnett's multiple tests, p(0% & 5%)= 0.1584, p(0% & 10%)=0.9998, p(0% & 15%)=0.8150, p(0% & 20%)=0.7275. Right, <FRET E> of Y27632 treated TS cells as a function of strain for parallel cells. n=54 for 0%, n=12 for 5%, n=16 for 10%, n=20 for 15%, n=20 for 20%. Dunnett's multiple tests, p(0% & 5%)=0.90, p(0% & 10%)=0.73, p(0% & 15%)=0.99, , p(0% & 20%)=0.63. (E) left, <FRET E> of Blebbistatin treated TS cells as a function of strain for orthogonal cells. n=28 for 0%, n=23 for 5%, n=10 for 10%, n=14 for 15%, n=9 for 20%. Dunnett's multiple tests, p(0% & 5%)=0.91, p(0% & 10%)=0.99, p(0% & 15%)=0.35, p(0% & 20%)=0.65. Right, <FRET E> of Blebbistatin treated TS cells as a function of strain for parallel cells. n=25 for 0%, n=14 for 5%, n=7 for 10%, n=4 for 15%. Dunnett's multiple tests, p(0% & 5%)=0.45, p(0% & 10%)=0.95, p(0% & 15%)=0.78. (F) left, <FRET E> of ck666 treated TS cells as a function of strain for orthogonal cells. n=27 for 0%, n=22 for 5%, n=14 for 10%, n=12 for 15%, n=10 for 20%. Dunnett's multiple tests, p(0% & 5%)= 0.99, p(0% & 10%)=0.98, p(0% & 15%)=0.4, p(0% & 20%)=0.50. Right, <FRET E> of ck666 treated TS cells as a function of strain for parallel cells. n=66 for 0%, n=26 for 5%, n=20 for 10%, n=7 for 15%, n=7 for 20%. Dunnett's multiple tests, p(0% & 5%)=0.99, p(0% & 10%)=0.99, p(0% & 15%)=0.94, , p(0% & 20%)=0.04. (G) applied strain-time steps on the PDMS chambers. (H) $<\Delta RFP>$ and $<\Delta RFP/A>$ as a function of strain for orthogonal cells. In $<\Delta RFP>$, n=14 for 5%, n=9 for 10%, n=6 for 15%, n=4 for 20%. In $<\Delta RFP/A>$, n=11 for 5%, n=6 for 10%, n=5 for 15%, n=4 for 20%. Sidak's multiple test was used. In $<\Delta RFP>$ p(5% & 10%)=0.9807 p(5% & 15%)=0.4323, p(5% & 20%)=0.8124, p(10% & 15%)=0.6922, p(10% & 20%)=0.9425, p(15% & 20%)=0.9822. In $<\Delta RFP/A>$ p(5% & 10%)=0.2021 p(5% & 15%)=0.0081, p(5% & 20%)<0.0001, p(10% & 15%)=0.5627, p(10% & 20%)=0.0035, p(15% & 20%)=0.1064 (I) $<\Delta RFP>$ and $<\Delta RFP/A>$ as a function of strain for parallel cells. In $<\Delta RFP/A>$, n=7 for 5%, n=8 for 10%, n=9 for 15%, n=5 for 20%. In $<\Delta RFP/A>$, n=8 for 5%, n=7 for 10%, n=9 for 15%, n=7 for 20%. Sidak's multiple test was used. In $<\Delta RFP>$ p(5% & 10%)=0.3557 p(5% & 15%)=0.6339, p(5% & 20%)=0.6569, p(10% & 15%)=0.9979, p(10% & 20%)>0.999, p(15% & 20%)>0.999. In $<\Delta RFP/A>$ p(5% & 10%)=0.9083 p(5% & 15%)=0.9466, p(5% & 20%)=0.8914, p(10% & 15%)>0.999, p(10% & 20%)=0.0035, p(15% & 20%)>0.999. Source data are provided as a Source Data file.

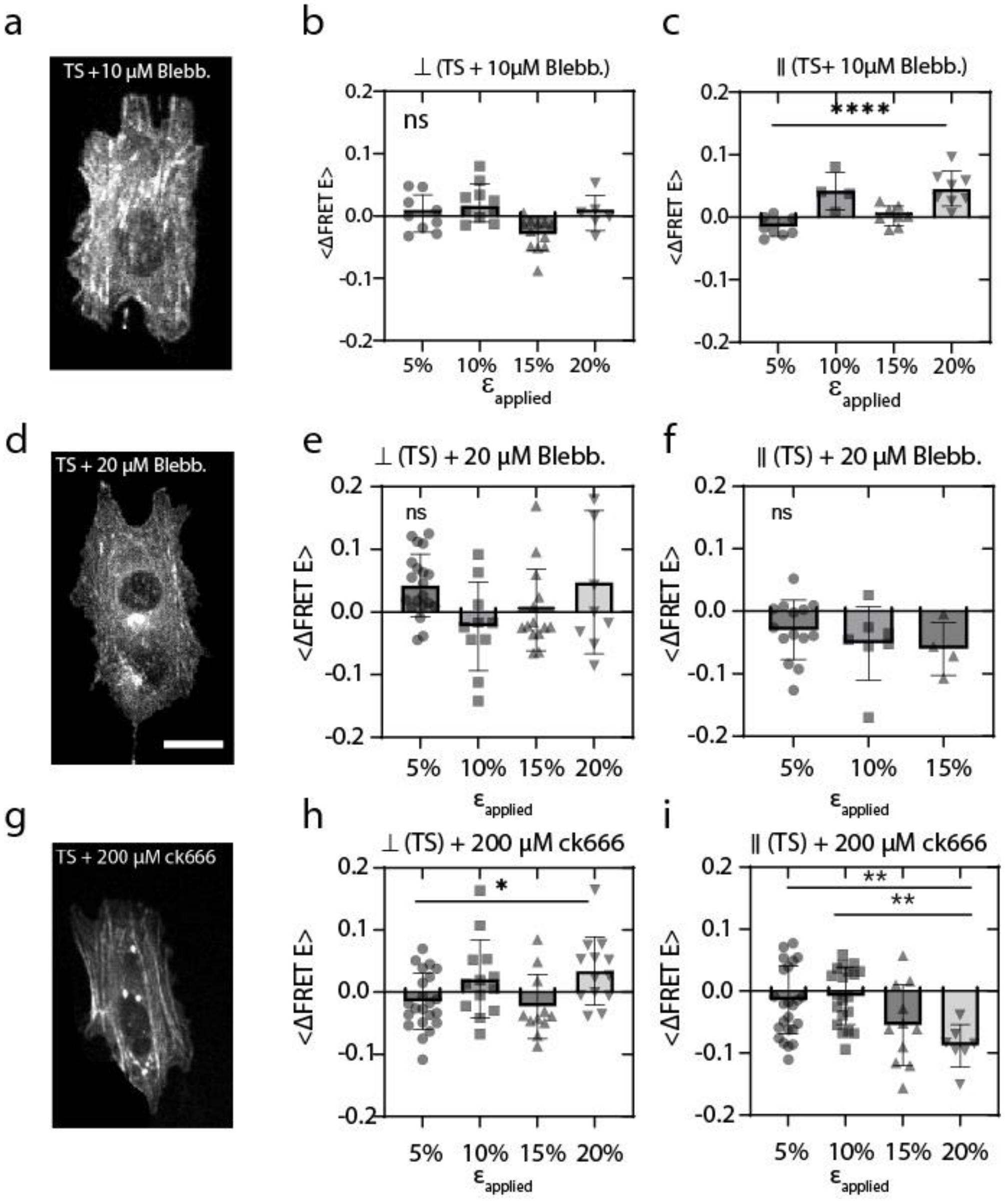

Supplementary Figure 13. Drug treatment controls for cell stretching. (a) TS cell treated with 10 μM Blebbistatin. (b) <FRET E> for TS cell treated with 10 μM Blebbistatin, orthogonal (n=9 for 5%, n=10 for 10%, n=11 for 15%, n=6 for 20%). One-way ANOVA test was used. p(5% & 10%)=0.5168, p(5% & 15%)=0.1178, p(5% & 20%)<0.999, p(10% & 15%)=0.003, p(10% & 20%)=0.6466, p(15% & 20%)=0.1743 (c) parallel (n=8 for 5%, n=4 for 10%, n=8 for 15%, n=8 for 20%). Tukey's significance test was used. p(5% & 10%)=0.0011, p(5% & 15%)=0.1178, p(5% & 20%)<0.999, p(10% & 15%)=0.003, p(10% & 20%)=0.6466, p(15% & 20%)=0.1743 (d) TS cell treated with 20 μM Blebbistatin. (e) <FRET E> for TS cell treated with 20 μM Blebbistatin, orthogonal (n=20 for 5%, n=10 for 10%, n=14 for 15%, n=9 for 20%). Tukey's significance test was used. p(5% & 10%)=0.0964, p(5% & 15%)=0.399, p(5% & 20%)=0.998, p(10% & 15%)=0.8116, p(10% & 20%)=0.1532, p(15% & 20%)=0.4763. (f) For parallel cells (n=14 for 5%, n=7 for 10%, n=4 for 15%). Tukey's significance test was used. p(5% & 10%)=0.6340, p(5% & 15%)=0.5471, p(10% & 15%)=0.9574. (g) TS cell treated with 200 μM ck666. (h) <FRET E> for TS cell treated with 200 μM ck666, orthogonal (n=22 for 5%, n=13 for 10%, n=11 for 15%, n=13 for 20%). Tukey's significance test was used. p(5% & 10%)=0.2203, p(5% & 15%)=0.9770, p(5% &

20%)=0.0483, p(10% & 15%)=0.1894, p(10% & 20%)=0.9193, p(15% & 20%)=0.0510. (i) 200 µM ck666 parallel cells (n=28 for 5%, n=20 for 10%, n=11 for 15%, n=7 for 20%) p(5% & 10%)=0.9743, p(5% & 15%)=0.1404, p(5% & 20%)=0.007, p(10% & 15%)=0.0885, p(10% & 20%)=0.005, p(15% & 20%)=0.5646. ns is non-significant. Source data are provided as a Source Data file. Scale bar is 10 µm.

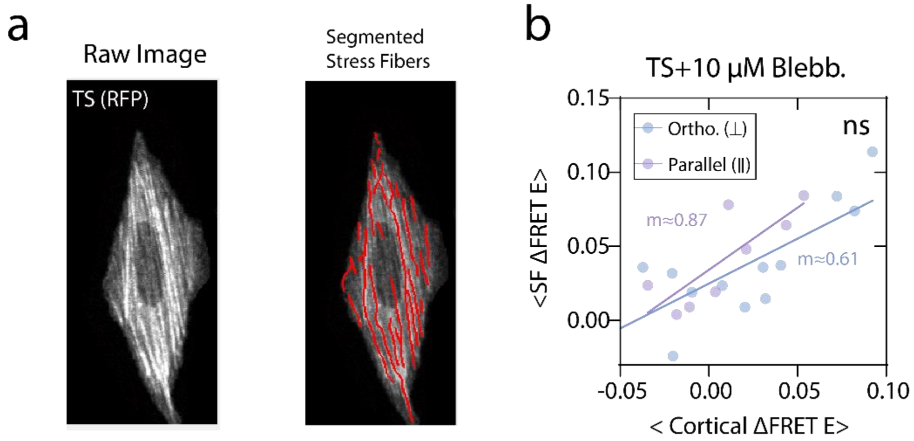

Supplementary Figure 14. Stress Fibers Segmentation. (A) Raw image of cells seeded on PDMS substrates. Red lines indicate segmented stress fibers to generate the SF and cortical actin mask. (B) Relative change in internal forces of stress fibers vs cortical actin. Regression lines with p-value=0.0019 for ortho., p-value=0.0150 for parallel (Material and Methods). Scale bar is 5 µm. Raw data are provided as a Source Data file.

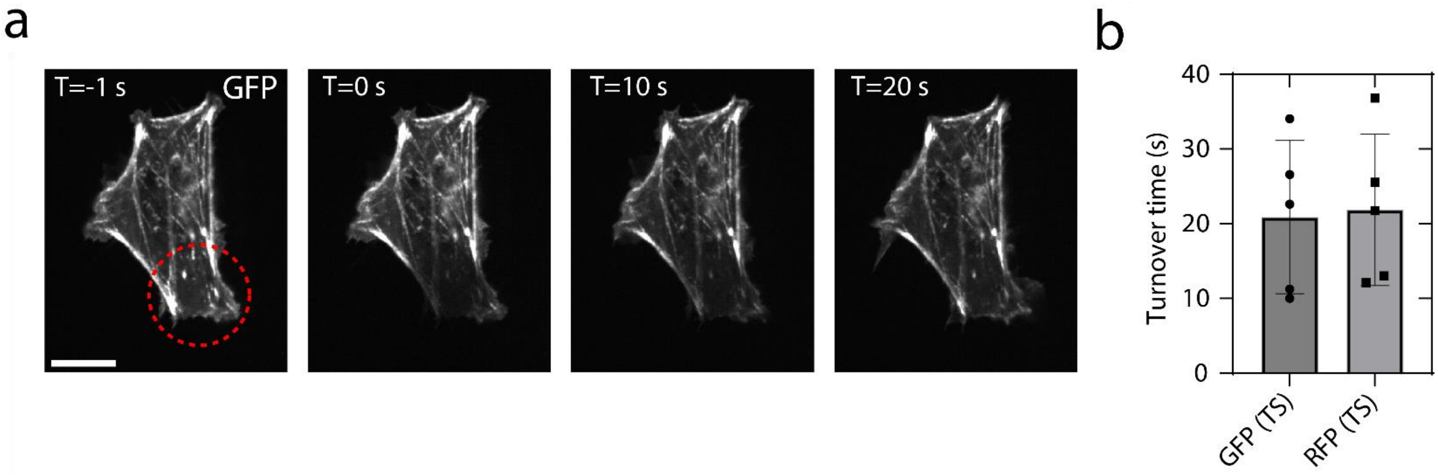

Supplementary Figure 15. Tension sensor turnover time scales by FRAPPA. (a) FRAPPA of the GFP and RFP of the tension sensor (b) RFP (n=5) and GFP (n=5) turnover timescales from exponential fit to the average intensity of frapped area. Red line indicates region for FRAPP. Scale bar is 10 µm. Source data are provided as a Source Data file.

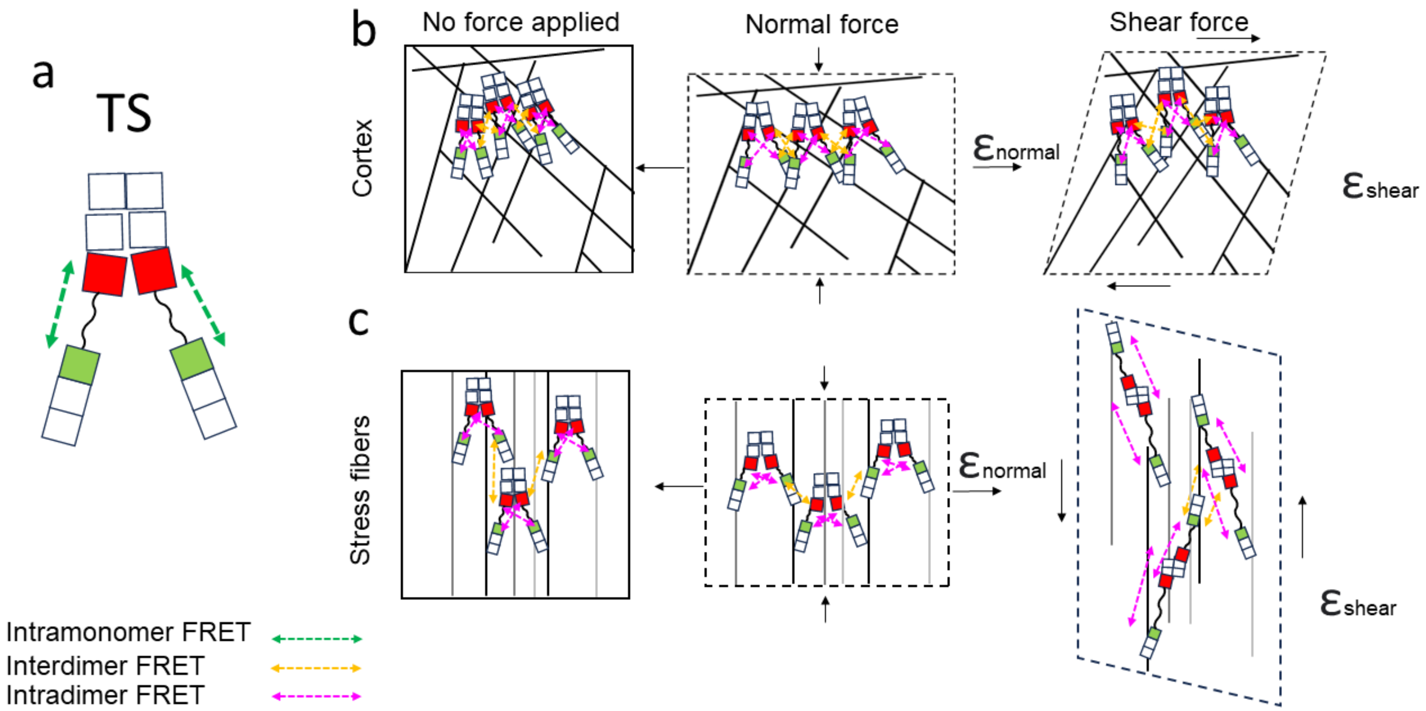

Supplementary Figure 16. Schematic of the tension sensor and associated intramonomer, interdimer and intradimer FRET under stretch. (a) dimeric tension sensor and intramonomer FRET within each monomer. (b) Tension sensors on cortical network of actin, deformed under normal and shear forces. (c) Tension sensors on stress fibers, deformed under normal and shear forces. Green arrows indicate the intramonomer FRET, not shown in b and c for clarity. Yellow arrows indicate the interdimer FRET, between individual TS. Magenta arrows indicate the intradimer FRET, between FRET constructs within a single TS. The diagram suggests higher inter/intradimer FRET on the cortical actin compared to the stress fibers, due to the crosslinked nature of the cortex and their closer proximity. We also show that on the stress fibers, normal forces can lead to higher interdimeric FRET because of the proximity of the TS, as well as higher intradimeric and compression FRET. Under shear forces, by the potential relative sliding of SF filaments, the dimerized TS is likely to elongate leading to a decrease in inter/intradimeric FRET as well as an increase in tension. On the cortex, with normal or shear forces, TS undergo tension, which also decreases their inter/intradimeric FRET.

Supplementary Video Legends

Supplementary Video 1. The FRET efficiency map (left) and traction stresses ($\vec{T}$, right) for a TS expressing U2OS cell on a circular pattern (no drug treatment control). Scale bar is 10 µm.

Supplementary Video 2. The FRET efficiency map (Left) and traction stresses ($\vec{T}$, right) for a TS expressing U2OS cell on a circular pattern treated with 10 nM Calyculin-A at 00:30 mins. Scale bar is 10 µm.

Supplementary Video 3. The FRET efficiency map (Left) and traction stresses ($\vec{T}$, right) for a TS expressing U2OS cell on a circular pattern treated with 10 µM Blebbistatin at 00:40 mins. Because of Blebbistatin inactivation with 488 nm laser, we incubate for 30 minutes before starting imaging again. Scale bar is 10 µm.

References

1. Bauer, A. *et al.* pyTFM: A tool for traction force and monolayer stress microscopy. *PLoS Comput. Biol.* **17**, 1–17 (2021).
2. Tambe, D. T. *et al.* Monolayer Stress Microscopy: Limitations, Artifacts, and Accuracy of Recovered Intercellular Stresses. *PLoS One* **8**, (2013).
