# Supplementary material for: Intracellular tension sensor reveals mechanical anisotropy of the actin cytoskeleton
Source: Nat Commun. 2023 Dec 4;14:8011. doi: 10.1038/s41467-023-43612-5 (PMC10695988; doi:10.1038/s41467-023-43612-5)
Supplement: Supplementary file 3 — Description of Additional Supplementary Files [file 41467_2023_43612_MOESM3_ESM.pdf]

Description of Additional Supplementary Files

Supplementary Movie 1. The FRET efficiency map (left) and traction stresses ($\vec{T}$, right) for a TS expressing U2OS cell on a circular pattern (no drug treatment control). Scale bar is 10 µm.

Supplementary Movie 2. The FRET efficiency map (Left) and traction stresses ($\vec{T}$, right) for a TS expressing U2OS cell on a circular pattern treated with 10 nM Calyculin-A at 00:30 mins. Scale bar is 10 µm.

Supplementary Movie 3. The FRET efficiency map (Left) and traction stresses ($\vec{T}$, right) for a TS expressing U2OS cell on a circular pattern treated with 10 µM Blebbistatin at 00:40 mins. Because of Blebbistatin inactivation with 488 nm laser, we incubate for 30 minutes before starting imaging again. Scale bar is 10 µm.
