# Supplementary material for: Intracellular tension sensor reveals mechanical anisotropy of the actin cytoskeleton
Source: Nat Commun. 2023 Dec 4;14:8011. doi: 10.1038/s41467-023-43612-5 (PMC10695988; doi:10.1038/s41467-023-43612-5)
Supplement: Supplementary file 7 — Reporting Summary [file 41467_2023_43612_MOESM7_ESM.pdf]

nature portfolio

Corresponding author(s): Michael Murrell

Last updated by author(s): Nov 8, 2023

Reporting Summary

Nature Portfolio wishes to improve the reproducibility of the work that we publish. This form provides structure for consistency and transparency in reporting. For further information on Nature Portfolio policies, see our Editorial Policies and the Editorial Policy Checklist.

Statistics

For all statistical analyses, confirm that the following items are present in the figure legend, table legend, main text, or Methods section.

n/a	Confirmed	
☐	☒	The exact sample size (*n*) for each experimental group/condition, given as a discrete number and unit of measurement
☐	☒	A statement on whether measurements were taken from distinct samples or whether the same sample was measured repeatedly
☐	☒	The statistical test(s) used AND whether they are one- or two-sided<br>*Only common tests should be described solely by name; describe more complex techniques in the Methods section.*
☒	☐	A description of all covariates tested
☒	☐	A description of any assumptions or corrections, such as tests of normality and adjustment for multiple comparisons
☐	☒	A full description of the statistical parameters including central tendency (e.g. means) or other basic estimates (e.g. regression coefficient) AND variation (e.g. standard deviation) or associated estimates of uncertainty (e.g. confidence intervals)
☐	☒	For null hypothesis testing, the test statistic (e.g. *F*, *t*, *r*) with confidence intervals, effect sizes, degrees of freedom and *P* value noted<br>*Give P values as exact values whenever suitable.*
☒	☐	For Bayesian analysis, information on the choice of priors and Markov chain Monte Carlo settings
☒	☐	For hierarchical and complex designs, identification of the appropriate level for tests and full reporting of outcomes
☐	☒	Estimates of effect sizes (e.g. Cohen's *d*, Pearson's *r*), indicating how they were calculated

Our web collection on statistics for biologists contains articles on many of the points above.

Software and code

Policy information about availability of computer code

Data collection	ANDOR iQ 3.6
Data analysis	Custom written MATLAB(R) version 2021b script was used for FRET analysis.<br>Particle image velocimetry is done in MATLAB using the open source software MatPIV 1.7.<br>FSegment (https://doi.org/10.1111/jmi.12593), a MATLAB(R) written software was used for segmenting SFs and cortical actin.<br>PyTFM (https://doi.org/10.1371/journal.pcbi.1008364) was used to calculate the internal forces from TFM.<br>ImageJ was for basic image preprocessing.

For manuscripts utilizing custom algorithms or software that are central to the research but not yet described in published literature, software must be made available to editors and reviewers. We strongly encourage code deposition in a community repository (e.g. GitHub). See the Nature Portfolio guidelines for submitting code & software for further information.

Data

Policy information about availability of data

All manuscripts must include a data availability statement. This statement should provide the following information, where applicable:

- Accession codes, unique identifiers, or web links for publicly available datasets
- A description of any restrictions on data availability
- For clinical datasets or third party data, please ensure that the statement adheres to our policy

Data that support plots and other findings within this manuscript are available from the corresponding authors upon reasonable request, because of the large FRET image data.

Research involving human participants, their data, or biological material

Policy information about studies with human participants or human data. See also policy information about sex, gender (identity/presentation), and sexual orientation and race, ethnicity and racism.

Reporting on sex and gender	N/A
Reporting on race, ethnicity, or other socially relevant groupings	N/A
Population characteristics	N/A
Recruitment	N/A
Ethics oversight	N/A

Note that full information on the approval of the study protocol must also be provided in the manuscript.

Field-specific reporting

Please select the one below that is the best fit for your research. If you are not sure, read the appropriate sections before making your selection.

[x] Life sciences   [ ] Behavioural & social sciences   [ ] Ecological, evolutionary & environmental sciences

For a reference copy of the document with all sections, see nature.com/documents/nr-reporting-summary-flat.pdf

Life sciences study design

All studies must disclose on these points even when the disclosure is negative.

Sample size	Sample size is determined by experimental feasibility with approximately 10 independent trails per experiment. This number was adjusted when deemed sufficient when samples statistics were not affected by changes in sample size.
Data exclusions	Data is only excluded if an unforeseen experimental effect occurs. For example, at 20 uM blebbistatin concentration and 20% strains, cells started to detach and therefore we excluded those experiments. Also, in cell micropatterning experiments, the cells that did not respond to Calyculin-A or blebbistatin treatments based on traction force measurements were excluded from the analysis.
Replication	Reproducibility was determined by repetition of experiments by different co-authors. Experiments were performed and replicated in weekly intervals.
Randomization	This is not relevant to our study. We treat each cell under determined strains as an individual event and we group cells using the strain for each cell.
Blinding	Blinding is not relevant to our study. We use a measurable quantity to define strain values in our experiments and use drug treatments as controls to confirm.

Reporting for specific materials, systems and methods

We require information from authors about some types of materials, experimental systems and methods used in many studies. Here, indicate whether each material, system or method listed is relevant to your study. If you are not sure if a list item applies to your research, read the appropriate section before selecting a response.

Materials & experimental systems

n/a	Involved in the study	
☐	☒	Antibodies
☐	☒	Eukaryotic cell lines
☒	☐	Palaeontology and archaeology
☒	☐	Animals and other organisms
☒	☐	Clinical data
☒	☐	Dual use research of concern
☒	☐	Plants

Methods

n/a	Involved in the study	
☒	☐	ChIP-seq
☒	☐	Flow cytometry
☒	☐	MRI-based neuroimaging

Antibodies

Antibodies used	anti-Paxillin (Abcam ab32084, Knockout validated); secondary Alexa Fluor 647 (Abcam ab150075); secondary Alexa Fluor 405 (Abcam ab175671); Alexa Fluor 640 Phalloidin (Life Technologies);
Validation	Antibodies were used based on the validation from the manufacturer (knockout validated).

Eukaryotic cell lines

Policy information about cell lines and Sex and Gender in Research

Cell line source(s)	U-2 OS HTB-96™ (from ATTC).
Authentication	None of the cell lines were authenticated.
Mycoplasma contamination	cell lines were tested and eliminated for mycoplasma by the manufacturer.
Commonly misidentified lines (See ICLAC register)	None.
